# Investigating the phase diagram-ionic conductivity isotherm relationship in aqueous solutions of common acids: hydrochloric, nitric, sulfuric and phosphoric acid

**DOI:** 10.1038/s41598-024-56552-x

**Published:** 2024-04-03

**Authors:** Hilal Al-Salih, Yaser Abu-Lebdeh

**Affiliations:** Energy, Mining, and Environment Research Centre, National Research Council of Canada, 1200 Montreal Road, Ottawa, ON K1A 0R6 Canada

**Keywords:** Acids, Aqueous electrolyte, Liquid solution structure, Phase diagram, Ionic conductivity, Hydrochloric acid, Sulfuric acid, Phosphoric acid, Nitric acid, Electrochemistry, Physical chemistry

## Abstract

The relationship between phase diagram features around the solid–liquid equilibrium region and ionic conductivity in aqueous solutions is not well understood over the whole concentration range as is the case for acidic aqueous solutions. In this work, we have studied the ionic conductivity (*κ*) as a function of molar fraction (*x*) and temperature (*T*) for four acid/water solutions namely, monoprotic hydrochloric acid (HCl) and nitric acid (HNO_3_), diprotic sulfuric acid (H_2_SO_4_) and triprotic phosphoric acid (H_3_PO_4_) along with their binary phase diagrams. The connection between the main features of the phase diagrams and the trends in the ionic conductivity isotherms is established with a new insight on the two pertinent dominant conductivity mechanisms (hopping and vehicular). Ionic conductivity at different temperatures were collected from literature and fitted to reported isothermal (*κ* vs. *x*) and iso-compositional (*κ* vs. *T*) equations along with a novel semi-empirical equation (*κ* = f (*x*, *T))* for diprotic and triprotic acids. This equation not only has the best fit for acids with different valency; but also contains four parameters, less than any other similar equation in literature. This work is one of few that advances the understanding of the intricate relationship between structure and ionic transport in various acidic aqueous solutions.

## Introduction

Numerous studies have shown that the kind of salt and solvent used, as well as their concentration and temperature, affect the structure of liquid electrolyte solutions^1^. Unfortunately, there is minimal information available on the nature of structure and ion transport throughout the whole concentration range of electrolyte solutions, particularly at high concentrations^2^. Nevertheless, the intricacies of structure and ion transportation within liquid electrolyte solutions, particularly at high concentrations, are not fully understood or explored^2^. This area was dubbed as one of the greatest unresolved issues in physical chemistry by Angel, highlighting the prevalent disappointment among scientists due to the absence of an efficient model or theory that could displace or expand the long-standing Debye–Huckel theory^3^. Additionally, it's been long recognized that ionic conductivity is significantly influenced by electrolyte concentration, notably in aqueous solutions^4,5^. An in-depth examination of the solution's composition, its influence on transportation throughout varying concentration levels, and its correlation with the phase diagram, might provide valuable insights.

There are primarily two models that attempt to define the electrolyte solution's structure and its association with ion transportation: (1) A disordered model which is based on the famous ionic atmosphere model that draws upon the Debye–Huckel theory for electrolyte solutions and, to an extent, the Gouy-Chapman theory of the electric double layer of colloids. However, it was found to be only applicable to very dilute concentrations (less than 10 mM) with limited success in its modifications^2,6,7^. (2) An ordered model where ions and solvents are organized in unit cells and ion transport happens via a ‘hopping mechanism’ into empty sites or 'holes', rather than a hydrodynamic mechanism for ion transport between unit cells similar to conductivity in defective ionic solids like ionic solids, glass, and polymer electrolytes^6–9^.

One of the main features of the conductivity isotherms (κ vs. x) is the maximum observed at mild concentrations. The maximum persists over a temperature range with sometime shifts to higher concentrations. Understanding these characteristics require a deeper examination of the solution's structure, how it affects transport over the whole concentration range, and how it relates to the phase diagram. Abu-Lebdeh et al. have been putting special emphasis on a relationship between features in the phase diagram and trends in conductivity isotherms. They found an outstanding correlation between the first eutectic point in phase diagrams and conductivity maximum in the conductivity isotherm for many aqueous and non aqueous solutions^10–13^. Abu-Lebdeh proposed a model for electrolyte solutions that connect the structure of the liquid to its ionic conductivity through the binary phases diagram. Briefly, it postulates that the microstructure of the liquid, above the liquidus line of the phase diagram, is a heterogeneous mixture similar to that of the solid state below the corresponding solidus line. The heterogeneous microstructures vary with composition into molten solvent-rich domain (molten eutectic-in-molten solvent), molten eutectic and salt-rich domain (molten eutectic-in molten solvate or molten eutectic-in molten salt). Each domain is made up of charge carries/solvent molecules formed from the fragmentation of the bulk solid structure from below the solidus line into its basic building units of ion pairs (IPs), ionic clusters (ICs) or solvent aggregates above the liquidus line. The charge carriers are kinetically-stable entities that continuously undergo a rapid dynamic exchange of ions and solvents and conduct ions in their respective domains through accessible free volume by overcoming energy barriers under the influence of an electric field. Changes in the type of charge carriers, free volume, energy barrier plays a key role in controlling ion transport throughout the compositional range with the ion conductivity mechanism changing from being dominated by vehicular mechanism to one that is dominated by ion hopping on crossing the eutectic composition. Acids though, are an exception to this as ion hopping exists and dominates even before crossing the eutectic composition which explains their relatively high conductivity at the molar fraction of the highest conductivity in the isotherm (x_max_).

In this work, we decided to focus exclusively on acid solutions for their superior conductivity when compared to salts or hydroxides We have gathered the binary acid/water phase diagrams and isothermal/iso-compositional conductivity data for four common acids (hydrochloric acid (HCl), sulfuric acid (H_2_SO_4_), phosphoric acid (H_3_PO_4_) and nitric acid (HNO_3_)), and highlight the common correlations and features. We analysed activation energy data derived from the Arrhenius equation to help us explain the trends in conductivity data. We then present 3D plots of conductivity versus molar fraction and temperature. Finally, we fit the data to different empirical and semi-empirical equations found in literature along with an enhanced equation that fits the data better than any other equation with four parameters; while other equations in literature contain at least five.

## Phase diagrams and room temperature conductivity isotherms

**Figure 1:**
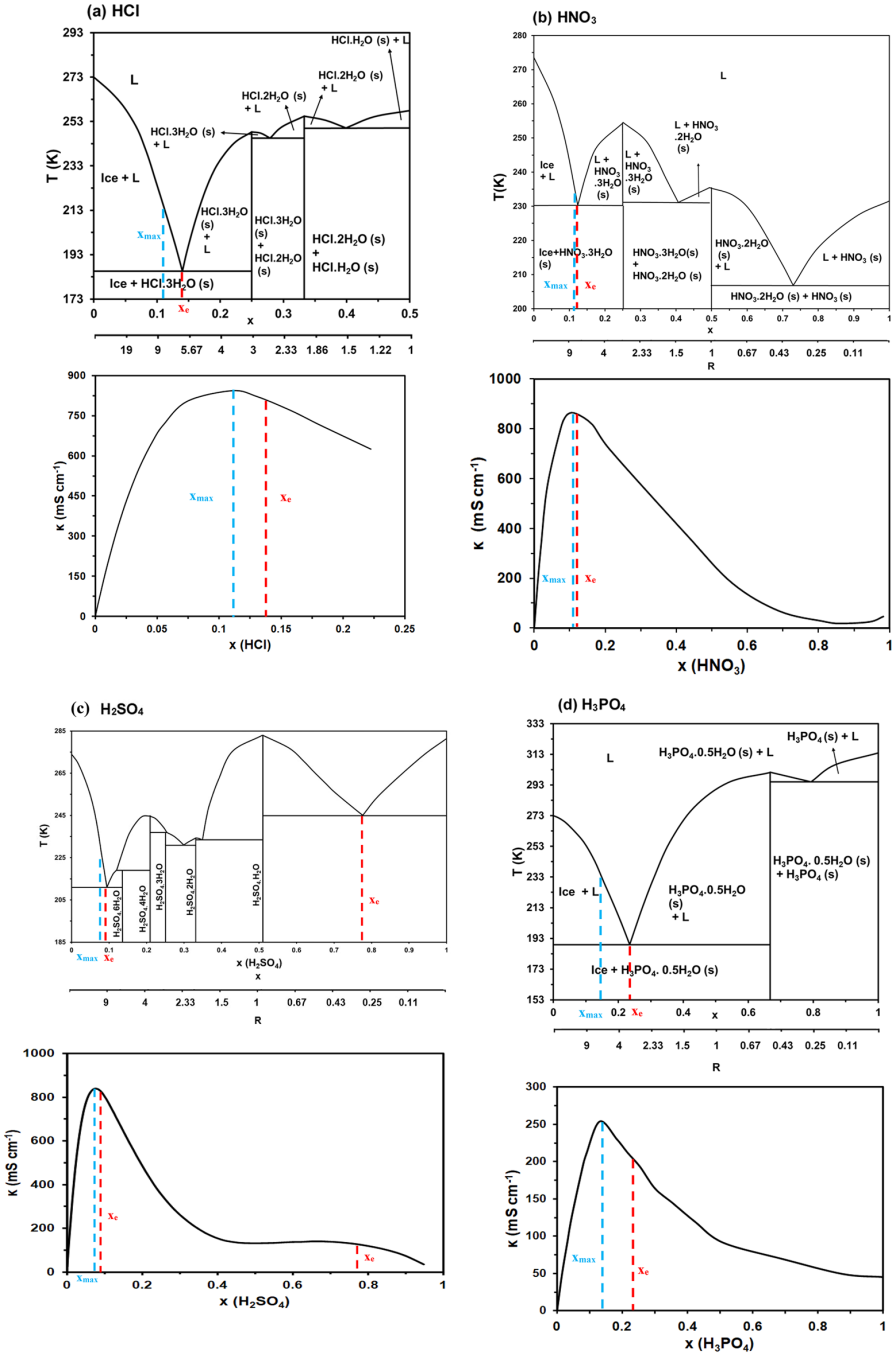
Phase diagram and room temperature ionic conductivity isotherms for (**a**) Hydrochloric acid (**b**) Nitric acid (**c**) Sulfuric acid (**d**) Phosphoric acid. Eutectic relevant to our discussion are labelled.

Figure 1 below present each acidic aqueous solutions room temperature conductivity isotherm below its respective phase diagram. Every region is properly labelled to show the phases existing at the respective *T* and *x*.

For HCl/water solution (Fig. 1a), the phase diagram can be divided into 3 simple phase diagrams (i.e., a phase diagram with one simple eutectic) separated by two solvates forming at *x* = 0.25 and 0.33 or *R* = 3 and 2, respectively. Eutectic points occur at x = 0.14, 0.28, and 0.4 at eutectic temperatures of 185 K, 246 K, and 250 K respectively. As was observed previously by our group for aqueous nitrate solutions, the first eutectic point (*x*_*eutectic*_) for this acid occurs within close proximity to the composition with the highest ionic conductivity on the room temperature conductivity isotherm (*x*_*max*_) which further solidifies this correlation^13^. In this case, *x*_*eutectic*_ = 0.14 and *x*_*max*_ = 0.11. The conductivity value at the *x*_*max*_ (*κ*_*max*_) is 844 mS cm^−1^_._

For HNO_3_/water solution (Fig. 1b), the phase diagram has the same features as HCl. It can be divided into 3 simple phase diagrams separated by two solvates forming at *x* = 0.25 and 0.5 or *R* = 3 and 1, respectively. Eutectic points occur at *x* = 0.12, 0.41, and 0.73 at eutectic temperatures of 230 K, 231 K, and 207 K respectively. Here, *x*_*eutectic*_ occurs exactly at *x*_*max*_ at 0.12 where *κ*_*max*_ is 860 mS cm^−1^_._ Nitric acid has the highest conductivity among the acids studied here and among all acids in general as well^14^.

For H_2_SO_4_/water solution (Fig. 1c), the phase diagram is more complex and a peritectic point at *x* = 0.14 at 319 K exists along side four eutectic points at *x* = 0.09, 0.30, 0.35, and 0.78 that occur at 211 K, 231 K, 235 K, and 245 K respectively. This phase diagram was not fully labelled to avoid overcrowding the figure. Instead, only the solvates forming were labeled. The prefixes here correspond to the *R* ratio. For example, monohydrate sulfuric acid means that a hydrate forms where *R* = 1. For this binary solution, *x*_*eutectic*_ (keep in mind this term is only used for the first eutectic) occurs at *x* = 0.09 which is again, close to *x*_*max*_ which occurs at *x* = 0.08 where *κ*_*max*_ = 836 mS cm^−1^.

For the weak acid H_3_PO_4_/water solution (rest are all strong acids) (Fig. 1d), the phase diagram is the simplest one. It can be divided into 2 simple phase diagrams separated by one solvate forming at *x* = 0.67 or *R* = 0.5. Eutectic points occur at *x* = 0.23, and 0.79 at eutectic temperatures of 189 K, and 295 K, respectively. Here, anomalous behavior is observed as *x*_*eutectic*_ (*x* = 0.23) occurs far from *x*_*max*_ (*x* = 0.14). A possible explanation for this behavior will be discussed in coming paragraphs. Notably, this acid has the weakest conductivity among the four acids studied here with *κ*_*max*_ = 255 mS cm^−1^ which is approximately one fourth of other acids *κ*_*max*_ values.

## Discussion

Table 1 lists all of the data used in the above description for phase diagrams and room temperature conductivity isotherms.

**Table 1:** Summary of the main features of the phase diagrams and ambient room temperature conductivity isotherms obtained.

Acid (solute)	Chemical structure	*x*_*eutectic*_	*T*_*eutectic*_ (K)	*x*_*max*_	*κ*_*max*_ (mS cm^−1^)	*x*_*solvate*_
Hydrochloric acid	HCl	0.14	185	0.11	844	0.25
		0.28	246			0.33
		0.40	250			0.5
Nitric acid	HNO_3_	0.12	230	0.12	860	0.25
		0.41	231			0.5
		0.73	207			
Sulfuric acid	H_2_SO_4_	0.09	211	0.08	836	0.14
		0.30	231			0.21
		0.35	235			0.25
						0.33
		0.78	245	0.71	137.5	0.51
Phosphoric acid	H_3_PO_4_	0.23	189	0.14	255	0.67
		0.79	295			

From Table 1, we notice that all three strong acids have very similar *κ *_*max*_ values of around 850 mS cm^−1^ except the weak acid H_3_PO_4_ that has approximately four times lower *κ*_*max*_. This is expected considering that strong acids dissociate completely into ions in water while weak acids partially dissociate. For *x*_*max*_, the trend is as follows: H_2_SO_4_ (0.08) < HCl (0.11) < HNO_3_ (0.12) < H_3_PO_4_ (0.14). It has been established that at least 10 molecules of water per one proton are required for optimal proton hopping (*R* = 10). higher number of protons would thus require more water molecules for optimal proton hopping^14,15^. This explains why H_2_SO_4_ x_max_ is lower than HCl or HNO_3_ *x*_*max*_. In the case of the triprotic weak H_3_PO_4_, *x*_*max*_ is higher than all acids and the trend is broken.

This is because H_3_PO_4_ has significantly lower dissociation constant (*Ka*) (6.9 × 10^–3^) which ultimately means less protons are free at the same *x* (despite the acid being triprotic) requiring less water for optimized hopping. This is better understood by Abu-Lebdeh’s model of liquid structure where the molten solvent-rich domain to molten eutectic-rich domain transition is delayed due to less break-up of the bulk solvent by the dissociated ions. The model accurately predicts the occurrence *x* at 0.1 for most liquid solutions.

From Table 2, a consistent trend emerges when analysing dissociation constants, pH values, and the room temperature isotherm infinite dilute region slope (*IDRS*) (Fig S.1 show a visualization of *IDRS*). Acids with lower *pKa* values demonstrate a steeper room temperature isotherm *IDRS* which arises from the increased ionic conductivity resultant from the larger number of protons in the solution. The steeper *IDRS* of the conductivity isotherm at room temperature provides a macroscopic confirmation of this increased ionic activity.

**Table 2:** Comparative analysis of various acids. This table presents each acid’s corresponding dissociation reactions, *pKa* values, and room temperature conductivity isotherm slope onset values.

Acid	dissociation reaction	*pKa* at 25 °C	*pH* (x = 0.01)	*IDRS*
Hydrochloric acid	HCl → Cl^−^ + H^+^	− 6.1 to − 7.0	∼0.26	18,203
Nitric acid	HNO_3_ → H^+^ + HNO_3_^−^	− 1.18 to 1.53	∼0.28	17,375
Sulfuric acid	H_2_SO_4_ → H^+^ + HSO_4_^−^	− 3.0 to − 3.5	∼0.21	21,675
	HSO_4_^−^ → H^+^ + SO_4_^2−^	1.96 to 2.04		
Phosphoric acid	H_3_PO_4_ → H^+^ + H_2_PO_4_^−^	2.12 to 2.17	∼1.35	3268
	H_2_PO_4_^−^ → H^+^ + HPO_4_^2−^	7.20 to 7.21		
	HPO_4_^2−^ → H^+^ + PO_4_^3−^	12.32 to 12.38		

The concept of proton transport in aqueous solutions, particularly for acids, is essentially governed by the Grotthuss mechanism. This unique mechanism delineates a rapid transfer of protons through a network of hydrogen bonds among water and hydronium ions, a phenomenon singularly possible due to the unique nature of the proton. The Grotthuss mechanism had been predominantly theoretical until recently, when a breakthrough study provided compelling experimental evidence supporting it. Utilizing advanced techniques such as dielectric spectroscopy, quasielastic neutron, and light scattering, along with ab initio molecular dynamic simulations, Popov et al.^16^ were able to observe and quantify the short 'jumps' of protons that characterise the Grotthuss mechanism. However, an intriguing finding from their study contradicts the conventional wisdom that the Grotthuss mechanism invariably enhances ionic conductivity. The high concentration of H_3_PO_4_ (85 wt%) means a lower '*R*' value (the ratio of water to acid molecules), leading to a dense molecular environment. This disrupts the hydrogen-bond network essential for the Grotthuss mechanism's efficiency. As a result, the mechanism's effectiveness declines with increasing concentration, particularly when *R* is below 10, leading to reduced conductivity due to the increased ionic correlations. Notably, the more gradual change in conductivity of phosphoric acid (H_3_PO_4_), both on the rise to the maximum concentration and in the subsequent decrease, compared to stronger acids like sulfuric acid (H_2_SO_4_) and nitric acid (HNO_3_), is potentially attributed to their higher pKa values, reflecting fewer dissociated protons with the increase in concentration. As the proton concentration changes more subtly in all regions, this results in a less abrupt change in conductivity. This theory not only expands our understanding of the Grotthuss mechanism but also lays the groundwork for further explorations of proton dynamics in acidic solutions. Vilciauskas et al.^17^ studied proton conductivity in pure phosphoric acid and also conduced that the two-step classical Grotthuss mechanism is not likely and that the high conductivity, that is coupled and 98% protonic, is due to an interplay between extended, polarized hydrogen-bonded chains and a frustrated hydrogen-bond network. This corroborates the existence of a heterogeneous structure as proposed by Abu-Lebdeh in his model of sub-micro domains where herein these can be a molten eutectic (molten hydrate of H_3_PO_4_.0.5H_2_O and molten pure H_3_PO_4_ ) and molten pure H_3_PO_4_ as per the phase diagram in Fig. 1d.

**Figure 2:**
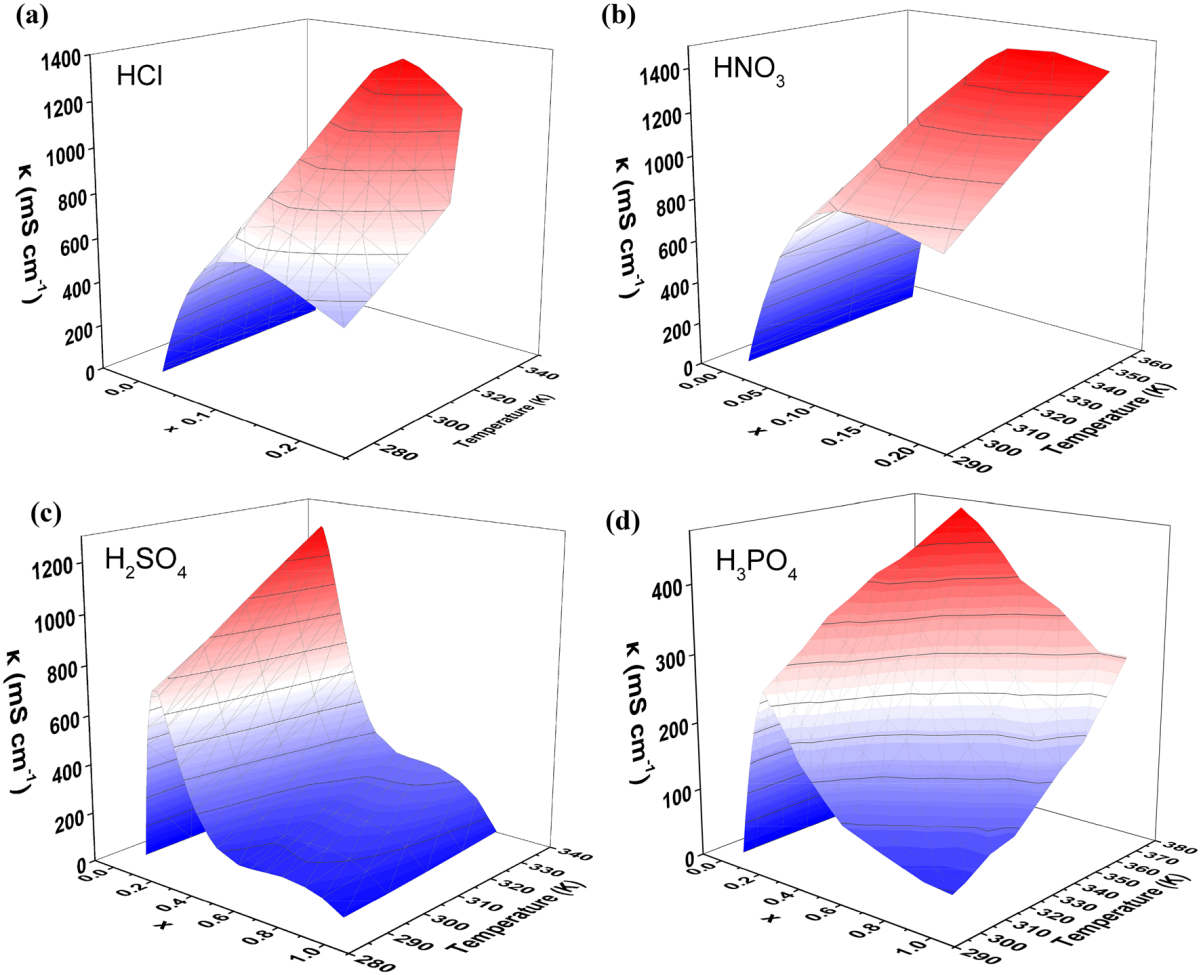
The variation of ionic conductivity (*κ*) with molar fraction (*x*) and temperature (*T*) for each of the aqueous acid solutions.

Figure 2 demonstrates how the ionic conductivity of the acidic aqueous solutions change with molar fraction and temperature. We can observe no shared trend in behavior across the four acid/water solutions other than the observation that *κ *_*max*_ increases with temperature. An interesting observation that can be made is that H_2_SO_4_/water solution is the only solution that exhibits two max points in conductivity, which are at x values close to its first and fourth eutectic point on the binary phase diagram. Further elucidation of the H_2_SO_4_/water solution's unique conductivity behavior is reported in a study by Das et al.^18^ who reveal intricate interplays between concentration, density, and viscosity. The density of sulfuric acid solutions incrementally increases with concentration, reaching a peak at 96.8 wt% H_2_SO_4_. Notably, the expansivities, demonstrate distinct concentration dependencies at temperatures like 298 and 243 K. This behavior aligns with structural changes within the H_2_SO_4_/H_2_O system, particularly in the 83–85 wt% and 92–94 wt% H_2_SO_4_ regions. These structural shifts correspond to the formations of H_2_SO_4_·H_2_O hydrate and the eutectic mixture between H_2_SO_4_·H_2_O and H_2_SO_4_, as evidenced in the conductance and viscosity isotherms and mirrored in the phase diagram^18^. Such correlations are not as apparent in the formations of other hydrates like H_2_SO_4_·2H_2_O, H_2_SO_4_·3H_2_O, and H_2_SO_4_·4H_2_O. The high concentration areas of these binary aqueous systems, where R is less than 2, exhibit a direct link between the phase diagram and viscosity characteristics, suggesting a maintained structural integrity in the solid H_2_SO_4_/H_2_O system within its liquid/supercooled liquid phase. This insight offers a deeper understanding of the conductivity peaks observed in sulfuric acid solutions, particularly in relation to their structural and phase transitions at specific concentrations.

Analysing all the graphs iso-compositionally, the conductivity of the solutions shows an upward trend with a rise in temperature with varying proportionality depending on x. In the dilute region a subtle enhancement in conductivity arises with an increase in temperature, whereas in regions with higher concentration near solubility, temperature increase significantly boost conductivity. This is primarily due to a greater number of ion pairs and clusters (IPs and ICs) in the concentrated areas compared to the diluted regions. As temperature rises, ion association decreases (dielectric constant decreases so association increase but the mobility of the ions increases)), resulting in a higher count of mobile "free" ions. As a result, conductivity in regions of high concentration is more sensitive to temperature changes. Additionally, temperature-induced expansion of the liquid augments its free volume and reduces the activation energy, thus improving ion mobility as explained below.

### Activation energy for ion conduction

In liquid electrolytes, solvated and un-solvated ions move by diffusing or hopping into empty sites or “free volume” when they acquire enough energy to overcome the energy barrier set up by neighboring ions or solvent molecules through ion-ion or ion–dipole interactions. This is the activation energy for ionic conductivity usually calculated from the Arrhenius relationship assuming a thermally-activated Eyring-like process.

1 $$\kappa = A_{o} exp\left( {\frac{{ - E_{a} }}{RT}} \right)$$

where ${A}_{o}$ is the pre-exponential factor, ${E}_{a}$ is the activation energy for ion conduction, *R* is the gas constant and *T* is the temperature in kelvin. *E*_*a*_ can then be divided into two components: *E*_*f*_ and *E*_*m*_ where *E*_*f*_ is the energy needed to form the free volume. It is mostly higher in ordered solids than disordered solids (e.g., glass) or ion solutions of molecular liquids because disordered structures have always higher number of defects “free volume” but require little expansion of ion pathways. *E*_*m*_ is the highest energy barrier for migration along the conduction path (the transition state for conduction).

It was reported by Abu-Lebdeh et al. and others that the activation energy for ion conduction in liquid electrolyte solutions decreases with an increase in concentration and goes through a minimum and then increases significantly. This was attributed to changes of the liquid structure from molten solvent-rich domain (high in free volume and ion solvent interactions) that decreases the barrier for ions to move to molten eutectic-rich domain (low in free volume and high in ion-ion interactions) that increase barrier for ions to move.

**Figure 3:**
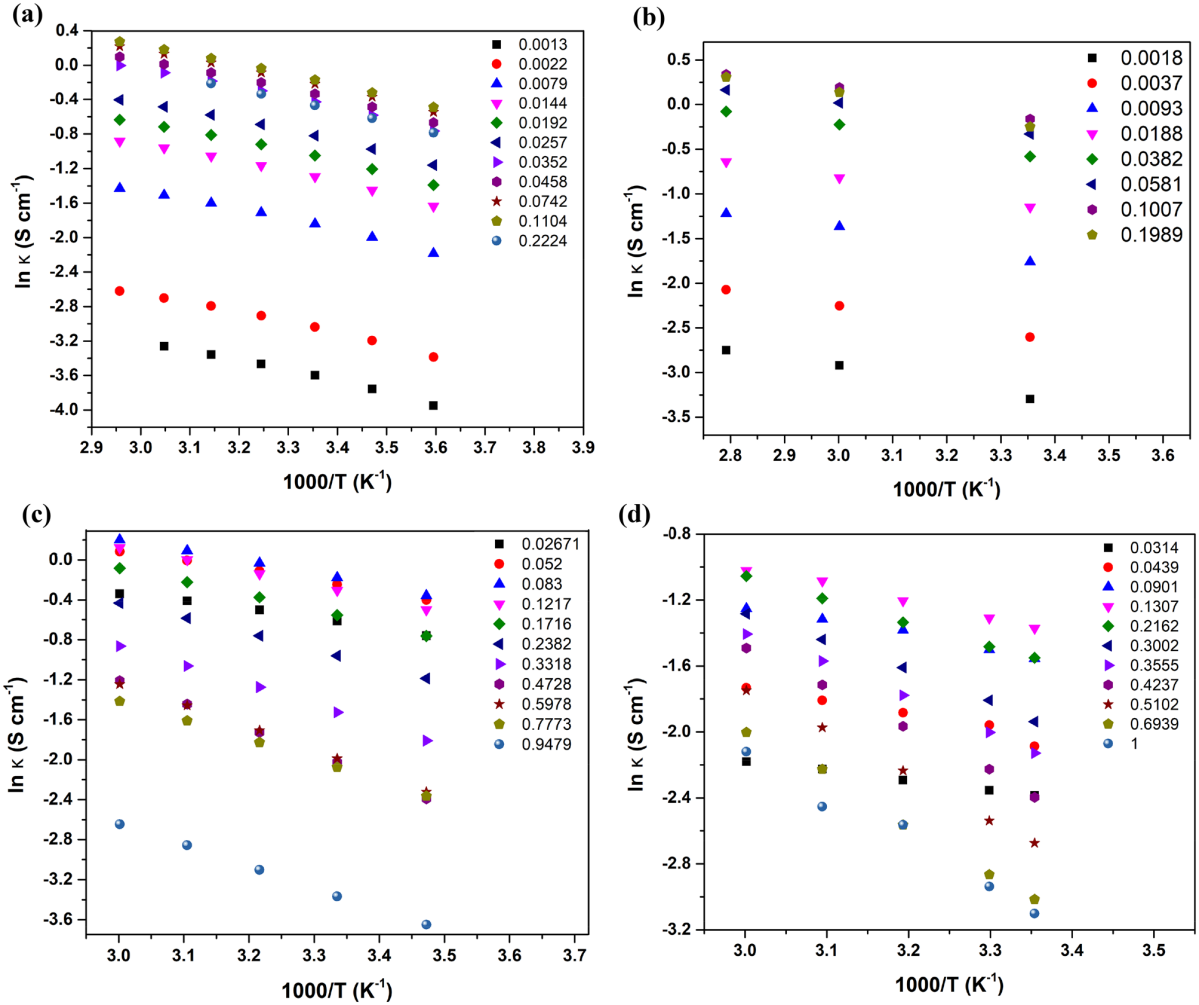
conductivity, ln (*κ*), of the different aqueous acid solutions as a function of temperature for (**a**) HCl (**b**) HNO_3_ (**c**) H_2_SO_4_, and (**d**) H_3_PO_4_.

To obtain the *E*_*a*_ and ${A}_{o}$ values, we must first linearize the *κ* vs *T*^−*1*^ data as in Fig. 3 below. The logarithm of conductivity (ln *κ*) as a function of temperature exhibits a similar linear trend for all electrolytes, suggesting their adherence to the traditional Arrhenius behavior as outlined in Eq. (1). Using the slopes and y-intercepts, we have calculated and graphed the *E*_*a*_ and ${A}_{o}$ data versus the *x* for each of the acids/water solutions.

While the Arrhenius model offers a robust framework for the linear temperature dependence of ionic conductivity observed in our data, it’s worth acknowledging alternative theoretical perspectives that address the complexities arising in highly concentrated solutions or near phase transition temperatures. The Vogel-Tammann-Fulcher (VTF) model is particularly relevant in scenarios where the Arrhenius behavior may not comprehensively describe the system's thermal dynamics. The VTF model is expressed in Eq. (2):

2 $$\kappa = \kappa_{o} exp\left( {\frac{ - B}{{T - T_{0} }}} \right)$$

where $\kappa$_0_ is the pre-exponential factor, *B* is a constant related to the activation energy for ionic movement, and *T*_*0*_ is the Vogel temperature, often interpreted as an ideal glass transition temperature. The VTF model accounts for the cooperative rearrangement of molecular segments, a significant factor in scenarios where the solvent structure and ion interactions exhibit dynamics akin to those in viscous or glassy states. Such conditions are frequently observed in highly concentrated aqueous solutions of acids, where conventional models like Arrhenius may not fully capture the nuanced behavior of ionic conductivity, especially at temperatures close to phase transitions or in highly structured solvent environments. Our current data align well with the Arrhenius model, indicating a predominantly linear temperature dependence of ionic conductivity.

**Figure 4:**
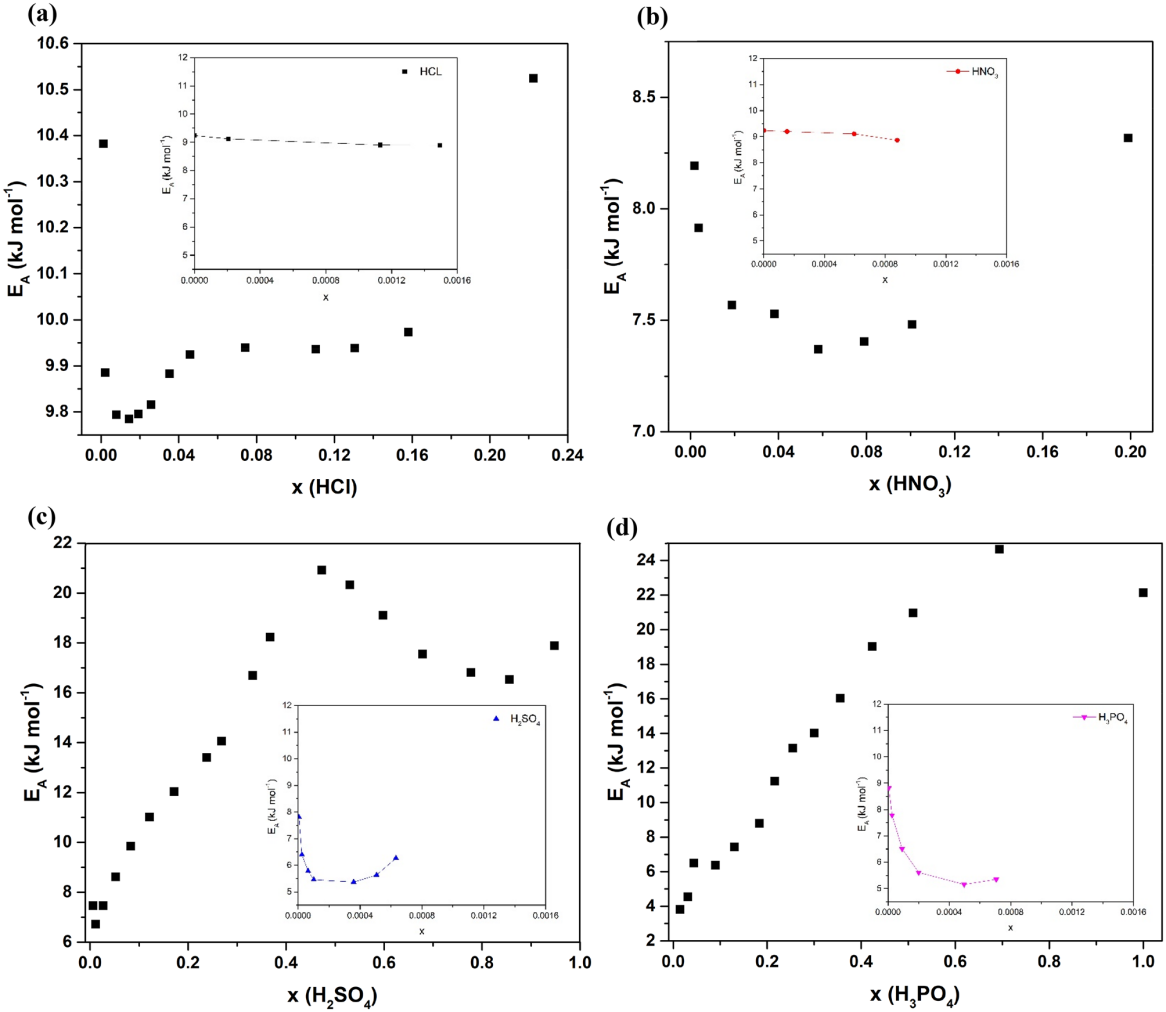
Activation energy for the different aqueous acid solutions as a function of molar fraction for (**a**) HCl (**b**) HNO_3_ (**c**) H_2_SO_4_, and (**d**) H_3_PO_4_.

Upon analysing the *E*_*a*_ vs *x* trends, one can certainly find a distinction between the behavior of HCl/HNO_3_ and that of H_2_SO_4_/H_3_PO_4_ acid/water solutions. The former acid/water solutions activation energy for the most part does not vary with concentration. To be precise, HCl/HNO_3_ activation energy fluctuations are contained with ± 1 kJ mol^−1^ which is less than half of the room temperature thermal energy of 2.47 kJ mol^−1^. Both of them have a minimum point on the curve but only HCl/water has a small plateau after the minimum point. On the other hand, H_2_SO_4_/water and H_3_PO_4_/water *E*_*a*_ vary significantly with concentration. These two solutions have a minimum at the very dilute region and then a significant ramp with increasing *x*. Their *E*_*a*_ reach a maximum at their most stable solvate formation points and then it drops back down. For H_2_SO_4_/water *E*_*a*_ goes back up after passing a eutectic point after the solvate formation while we do not have enough data points between *x* = 0.7 and *x* = 1 for the H_3_PO_4_ solution, we expect similar behavior in this region. Also, we observe that the H_2_SO_4_/water solution has a minimum point very close to infinite dilution and we do not have enough data points near infinite dilution for H_3_PO_4_/water solution. To help us fill this gap, Ivanov et al.^14^ has reported on *E*_*a*_ versus *x* for different acid/water solutions including H_2_SO_4_, H_3_PO_4_, and others at concentrations close to infinite dilution. Their results plotted in the inset plots in Fig. 4. yet again, demonstrate that the two solutions share the same behavior. Specifically, both solutions have a minimum point close to *x* = 0.0005 where the activation energy drops from 9.6 kJ mol^−1^ at infinite dilution to slightly above 5 kJ mol^−1^_._ At high *x* values, both solutions’ *E*_*a*_ increases to more than double the *E*_*a*_ at infinite dilution. Ivanov attributed this to the polymerization affect that happens within their structure at high concentration due to strong hydrogen bonding involving undissociated protons.

**Figure 5:**
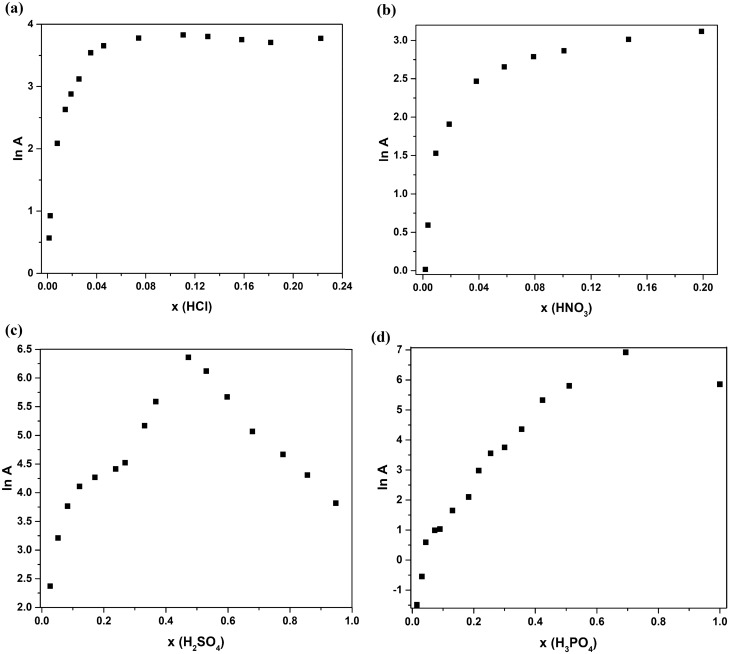
Pre-exponential factor for the different aqueous acid solution as a function of molar fraction.

Figure 5 depicts how *A* changes with *x*. *A* can be related to conductivity when there are no energy barriers to the movement of charge carriers (i.e., when E_a_ = 0 or T is infinitely large) and often related to jump frequency as described in the following equation^19,20^:

3 $$A = \gamma \, \lambda^{2} \Gamma$$

where *γ* is a geometric parameter that includes the number of close jump sites, *λ* is the jump distance and *Γ* is the jump frequency^20^. Here, acid solutions share the same trend here where *A* increases sharply in the dilute region and then plateaus at *x* > 0.08. H_2_SO_4_/water and H_3_PO_4_/water solutions also behave similarly beyond the plateau. For both solutions *A* increase sharply until hitting a maximum around the solvate forming molar fraction. Upon passing the maximum, drastic drop happens in the case of H_2_SO_4_ solution compared to a more gradual drop in the case of H_3_PO_4_ solution.

Numerous attempts have been made to find an empirical or semi-empirical equation that predicts *κ* at different *x* and *T* for aqueous electrolytes. In fact, the authors have recently proposed an equation (Eq. 3) that contains the least parameters (just two) among all other equations found in literature^13^. This equation was first tested against *κ* versus *x*-*T* data for four different aqueous nitrates solutions. To asses the validity of this equation, we have computed the coefficient of determination, *R*^2^, for the equation fitted curve against the nitrates solutions data. The equation proved to be of decent fit with *R*^2^ ≥ 95% which is not as solid as the *R*^2^ values for the other empirical equation (Eq. 5–6) reported in literature when fitted against certain solutions^21,22^. where they report *R*^2^ > 99% but using at least 3 additional parameters.

The oversimplification of the data fitting into a two-parameter equation could be the primary reason for observed over/under estimation. However, having only two parameters presents multiple advantages that enhance its usefulness in practice. Firstly, the model's simplicity makes it readily understandable and easier to interpret compared to models with more parameters. This clarity is especially beneficial for researchers and practitioners needing a direct but efficient predictive tool for conductivity in various scenarios. Secondly, the reduced number of parameters in our model lessens the chance of overfitting, ensuring better generalization to new, unexplored data. This feature is crucial for a model aiming to be useful across an extensive array of electrolytes.

In order to put this two-parameter equation to test, we decided to fit it against the acidic aqueous solutions in this study. Figure S.5 in the SI of this work shows the 3D figures of the fittings for the different acids. The *R*^2^ values are reported in Table 2. We can immediately realize that this equation only fits the monotropic acids well (*R*^2^ > 95%). However, it does not fit well for H_2_SO_4_/water and H_3_PO_4_/water solutions’ data. We can also notice that Eq. 5 and Eq. 6 also do not fit data for non monotropic acids well. This observation was less pronounced in the case of nitrates but we have also noticed that divalent nitrates had the weakest fit relative to the monovalent solutions^13^. We can conclude that Eq. 3 works best for monovalent aqueous solutions.

Herein, we propose a simple modification to Eq. 3 by introducing two additional parameters to come up with Eq. 4.

4 $$\kappa = AT^{B} x^{C} exp\left( {\frac{ - Dx}{T}} \right)$$

As can be inferred from Table 3, Eq. (4) has significantly improved *R*^2^ values when compared to Eq. (2). It also proves to have the highest average *R*^2^ when applied against all the acidic aqueous solutions data while still maintaining the least number of parameters. This equation provides almost perfect fit to monotropic acids and enhances *R*^2^ for H_2_SO_4_/water solution. H_3_PO_4_/water solution remains the trickiest solution to fit. Table 4 below lists the fitting parameters for the four different acid solutions.

**Table 3:** * R*^2^ values for each acid/water solution when fitted against different equations from literature and this work.

Equation	Acid	*R*^2^ (%)	Avg.* R*^2^ (%)	Reference
$\kappa = ATxexp\left( {\frac{ - Bx}{T}} \right)\quad\quad\quad\left( 3 \right)$	HCl	95.7	92.6	^13^
	HNO_3_	98.6		
	H_2_SO_4_	91.5		
	H_3_PO_4_	84.6		
$\kappa = AT^{B} x^{C} exp\left( {\frac{ - Dx}{T}} \right) \quad\quad\quad\left( 4 \right)$	HCl	99.5	96.6	This work
	HNO_3_	99.7		
	H_2_SO_4_	94.5		
	H_3_PO_4_	92.7		
$\kappa = xexp\left( {A + BT^{0.5} Cx^{0.5} } \right) + D + ET^{0.5} + Fx^{0.5}\quad\quad\quad\left( 5 \right)$	HCl	99.5	82.7	^21^
	HNO_3_	99.7		
	H_2_SO_4_	38.9		
	H_3_PO_4_	92.6		
$\kappa = \left( {AT + B} \right)x^{C} exp\left( {\frac{ - Dx}{{T - E}}} \right)\quad\quad\quad\left( 6 \right){ }$	HCl	99.9	96.0	^22^
	HNO_3_	99.8		
	H_2_SO_4_	94.1		
	H_3_PO_4_	90.3

**Table 4:** Fitted parameters and coefficient of determination for Eq. (3) for the four different aqueous acid solutions.

HCl	0.0056	2.65	0.97	2813	99.5
HNO_3_	0.20	2.10	1.06	3543	99.7
H_2_SO_4_	0.00052	2.96	0.76	2709	94.5
H_3_PO_4_	0.00014	2.82	0.71	1010	92.7

## Conclusion

We have studied the ionic conductivity data of different acidic aqueous solutions over a wide concentration and temperature ranges. To summarize, we were able to further corroborate our previous observation on the correlation between first eutectic point in binary phase diagrams and the point of highest conductivity in the room temperature conductivity isotherms^10,11,13^. Weak acid H_3_PO_4_ aqueous solution exhibited lower conductivity values and this is owing to its low dissociation constant. We have also noticed a distinction in the conductivity behavior after crossing the point of highest conductivity depending on the valency of the acids’ cation. This is most likely related to the difference in proton concentration at the same acid molar fraction. We analyzed trends in activation energy and pre-exponential factor changes with molar fraction. Both HCl/water and HNO_3_/water show subtle changes in activation energy across the concentration range while H_2_SO_4_/water and H_3_PO_4_/water show a minimum close to infinite dilution followed by an aggressive increase with increasing molar fraction until the solvate formation concentration. Finally, we try to fit the 3D curves of conductivity versus molar fraction and temperature against different literature equations to assess their applicability to the acids/water solutions. All equations fit the monotropic acids better and hence, we propose a modified equation with four total parameters that prove to have the best fit to the data. The supplementary information section of this paper contains all figures displaying the fit quality of all the equations against the four different acid/water solutions.

### Supplementary Information

Supplementary Information.

## Data Availability

Phase diagrams for HCl, HNO_3_, H_2_SO_4_, and H_3_PO_4_ solutions in water were obtained from Boryak et al.^23^, Tizek et al.^24^, Kinnibrugh et al.^25^, and Corti et al.^26^ respectively. The data from the four binary phase diagrams were digitized using Automeris software. Ionic conductivity data for HCl, HNO_3_, H_2_SO_4_, and H_3_PO_4_ solutions in water over the whole concentration range, and at temperatures ranging from 10 to 100 °C were collected from Owen et al.^27^, Spencer et al.^28^, Darling^29^, and Chin et al.^30^ respectively. The data for each acid were obtained in raw numerical format and not digitized from figures. HCl conductivity data was obtained as specific conductance so each data point was multiplied by its corresponding normality. For the rest of the acids, no further calculations were required. The units used were unified to be always in mS cm^−1^. Figure 1 present raw data as presented in the literature The remainder of the figures in text were generated after analyzing the data in MS excel and OriginPro. All files are available upon reasonable request.
