# Supplementary material for: Investigating the phase diagram-ionic conductivity isotherm relationship in aqueous solutions of common acids: hydrochloric, nitric, sulfuric and phosphoric acid
Source: Sci Rep. 2024 Apr 3;14:7894. doi: 10.1038/s41598-024-56552-x (PMC10991257; doi:10.1038/s41598-024-56552-x)

**Supplementary information**


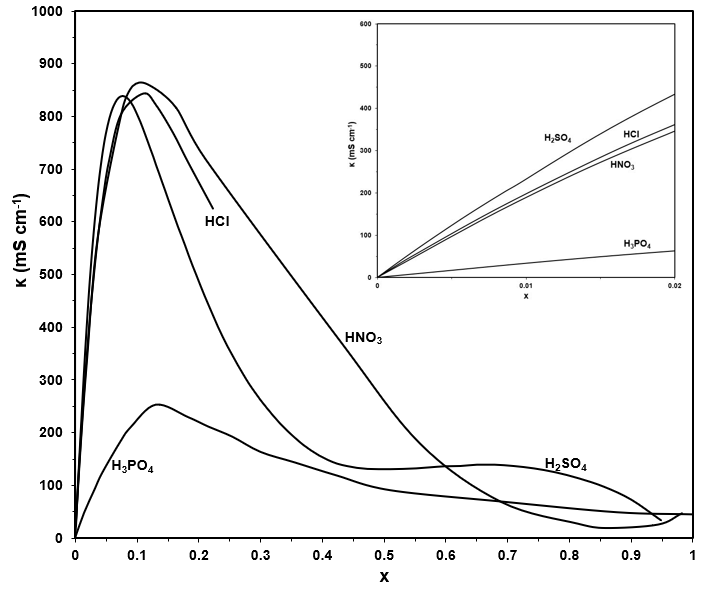
**Fig S.1:** Room temperature conductivity isotherms of the different acids with in-set graph of dilute region isotherms

**Fig S.2:** Fitting of eq. 2 against k vs x-T data for the four acidic aqueous solution


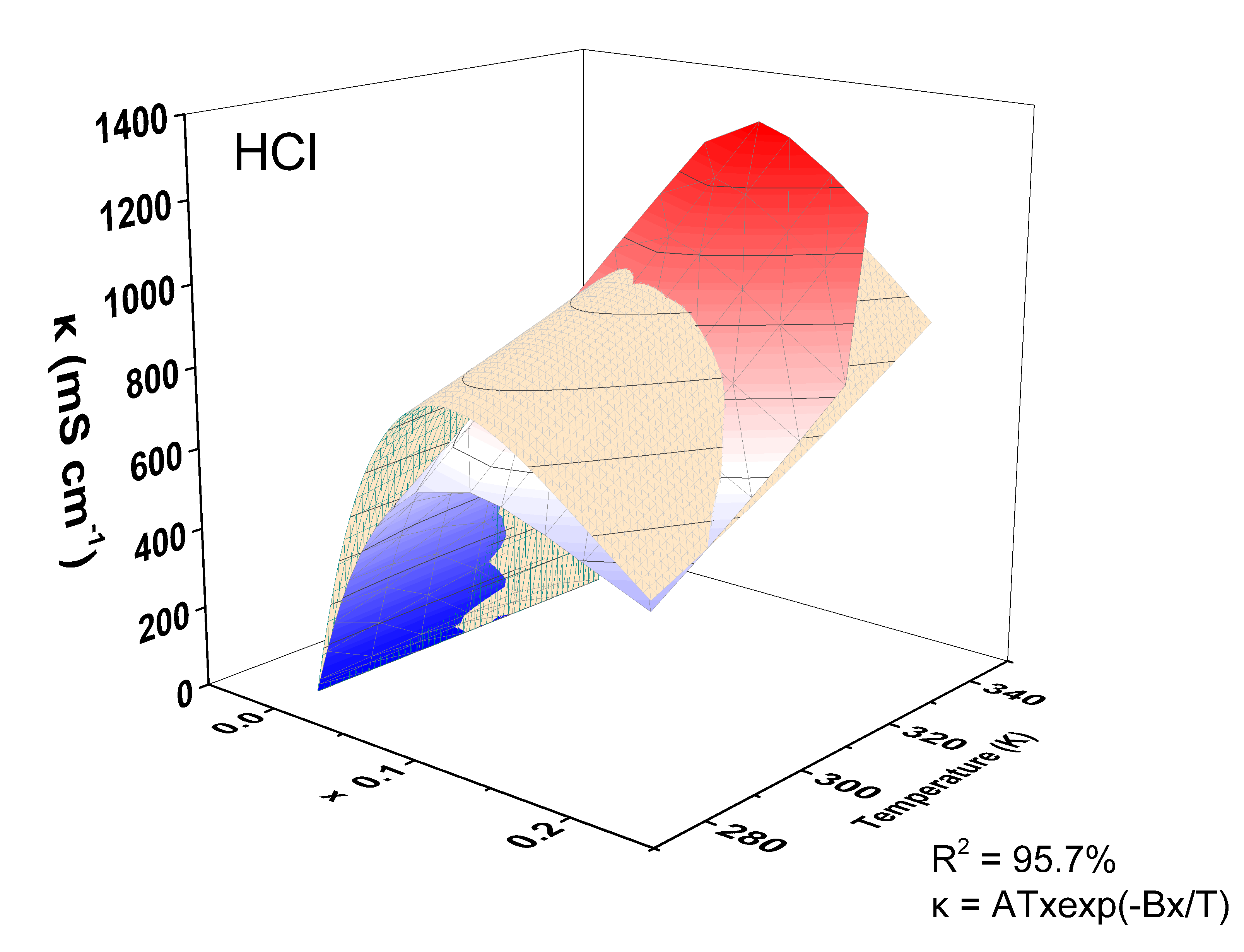

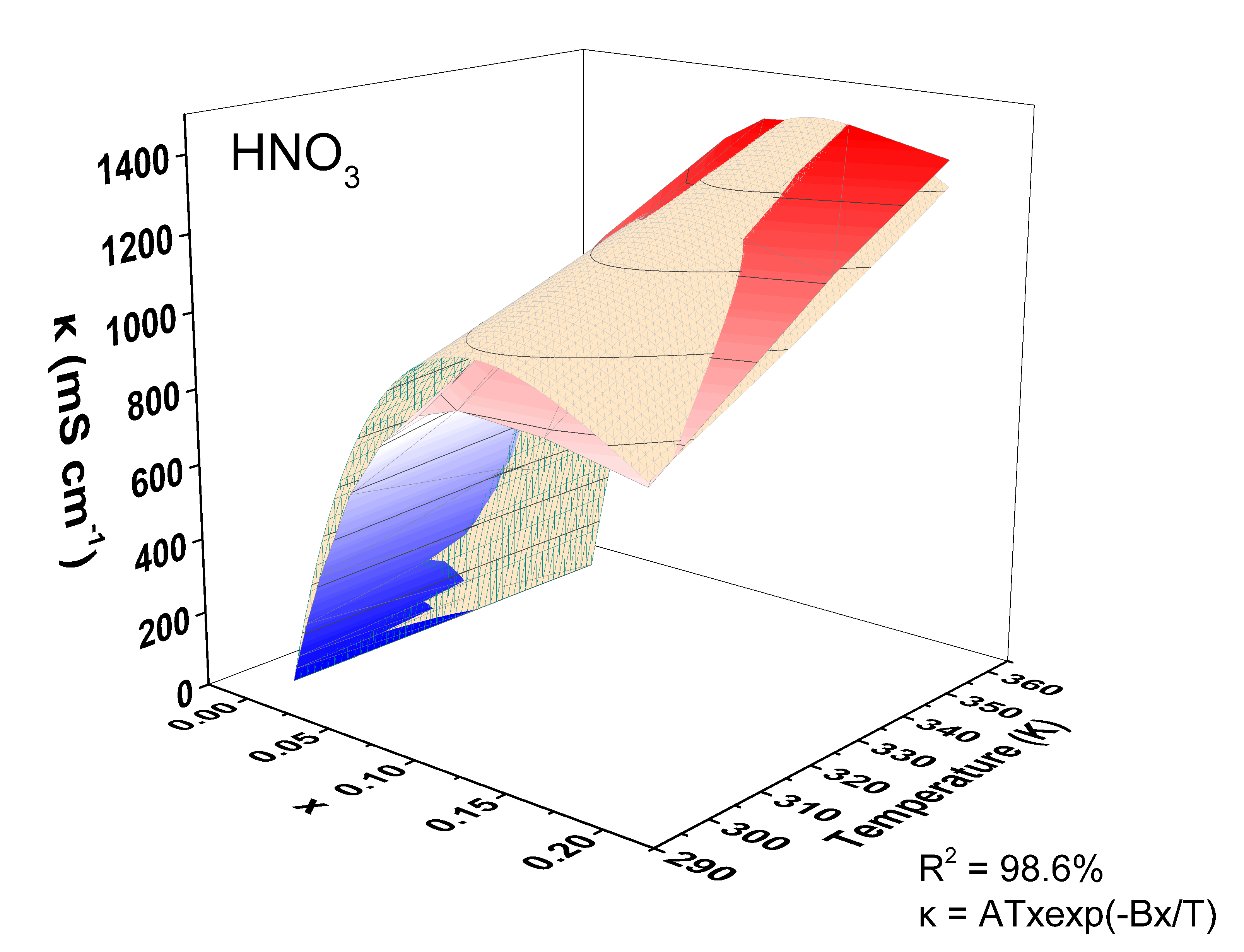

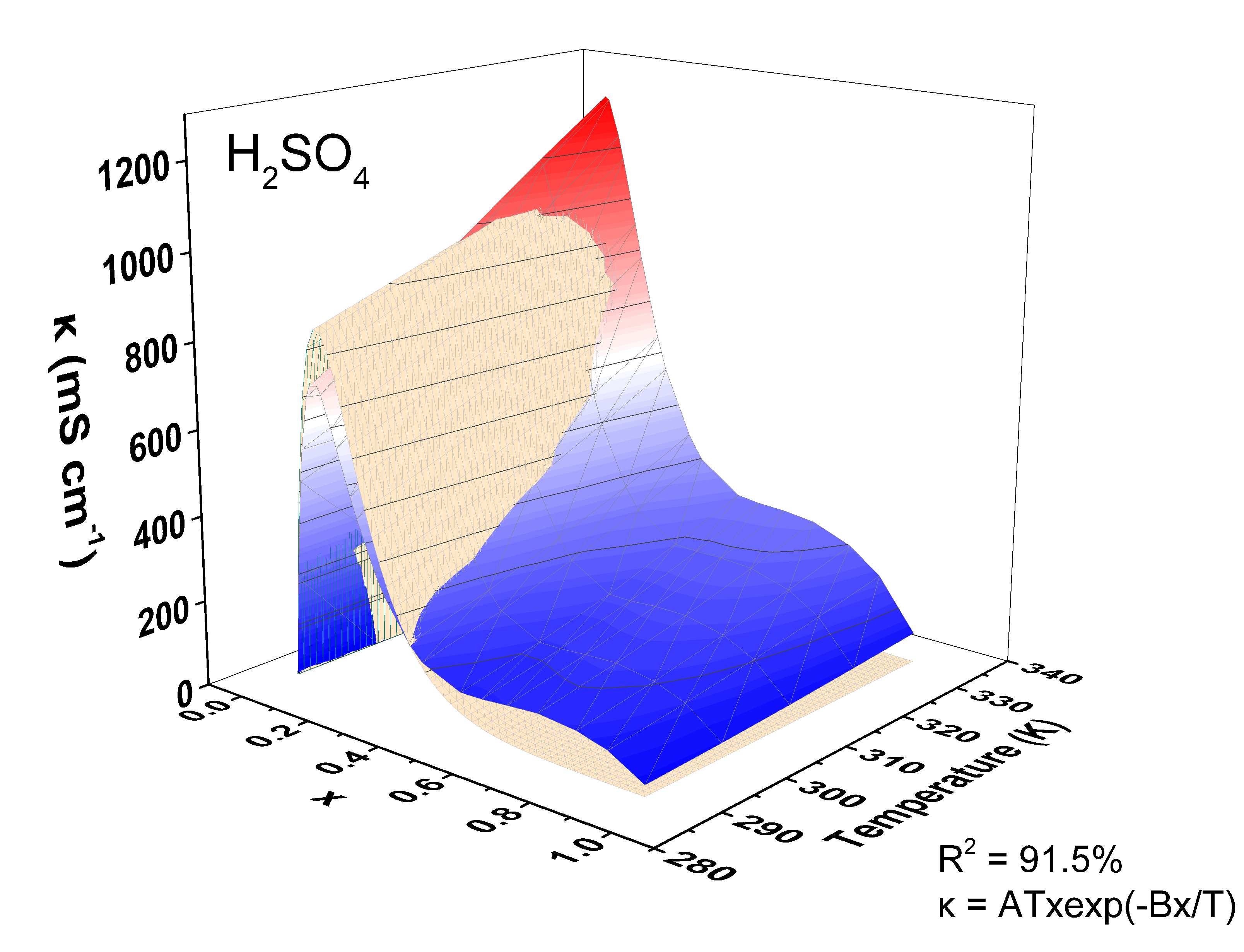

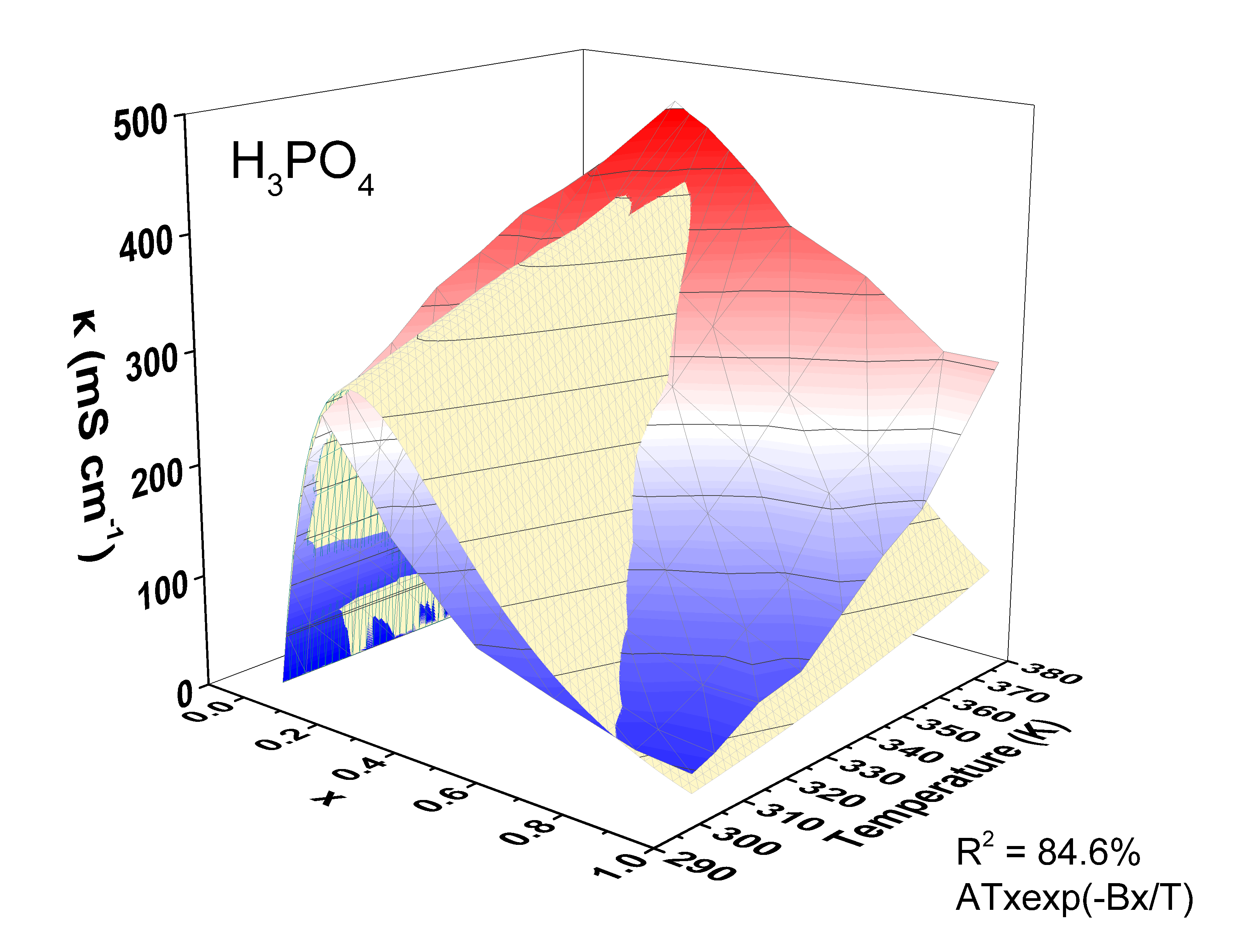


**Fig S.3:** Fitting of eq. 3 against k vs x-T data for the four acidic aqueous solution


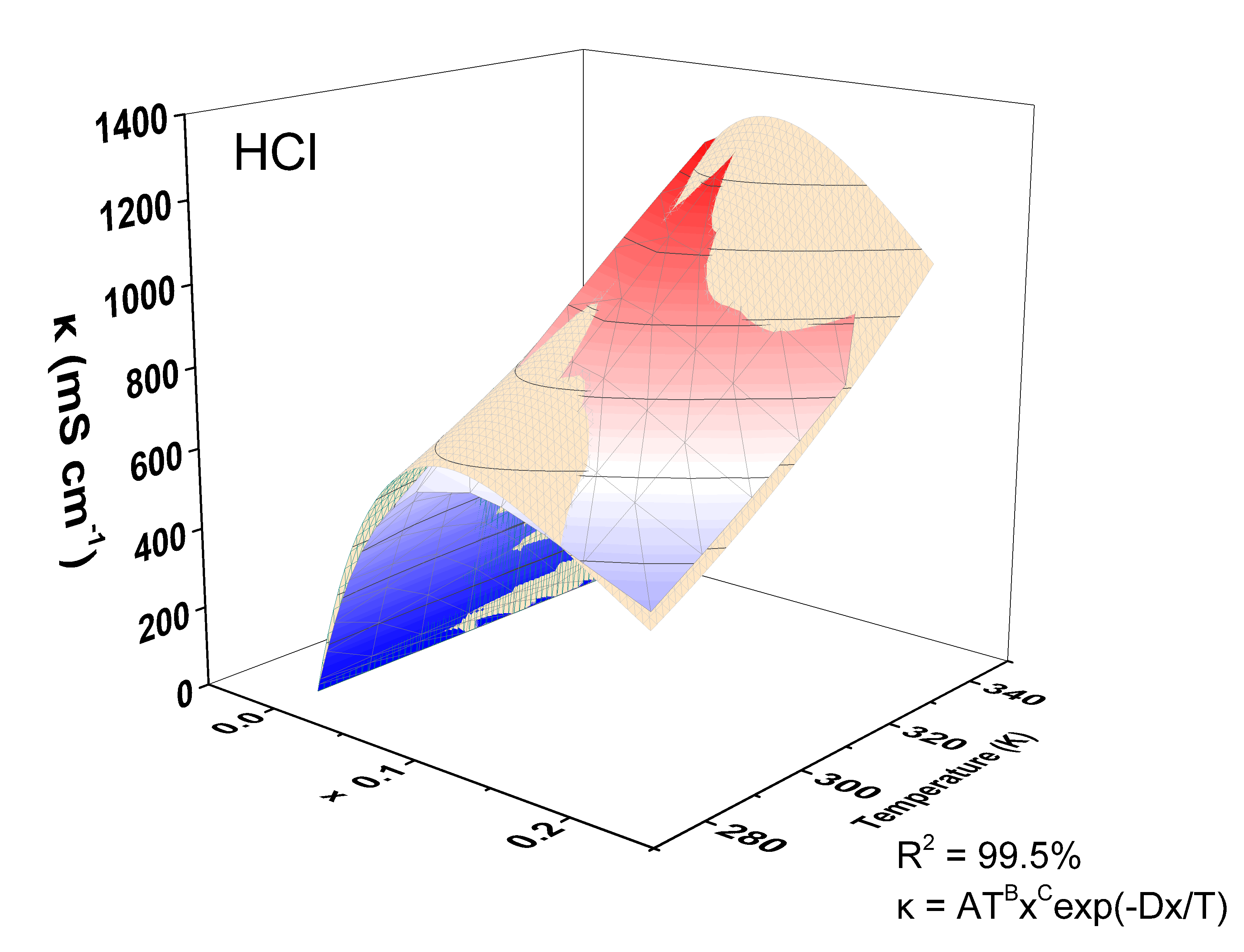

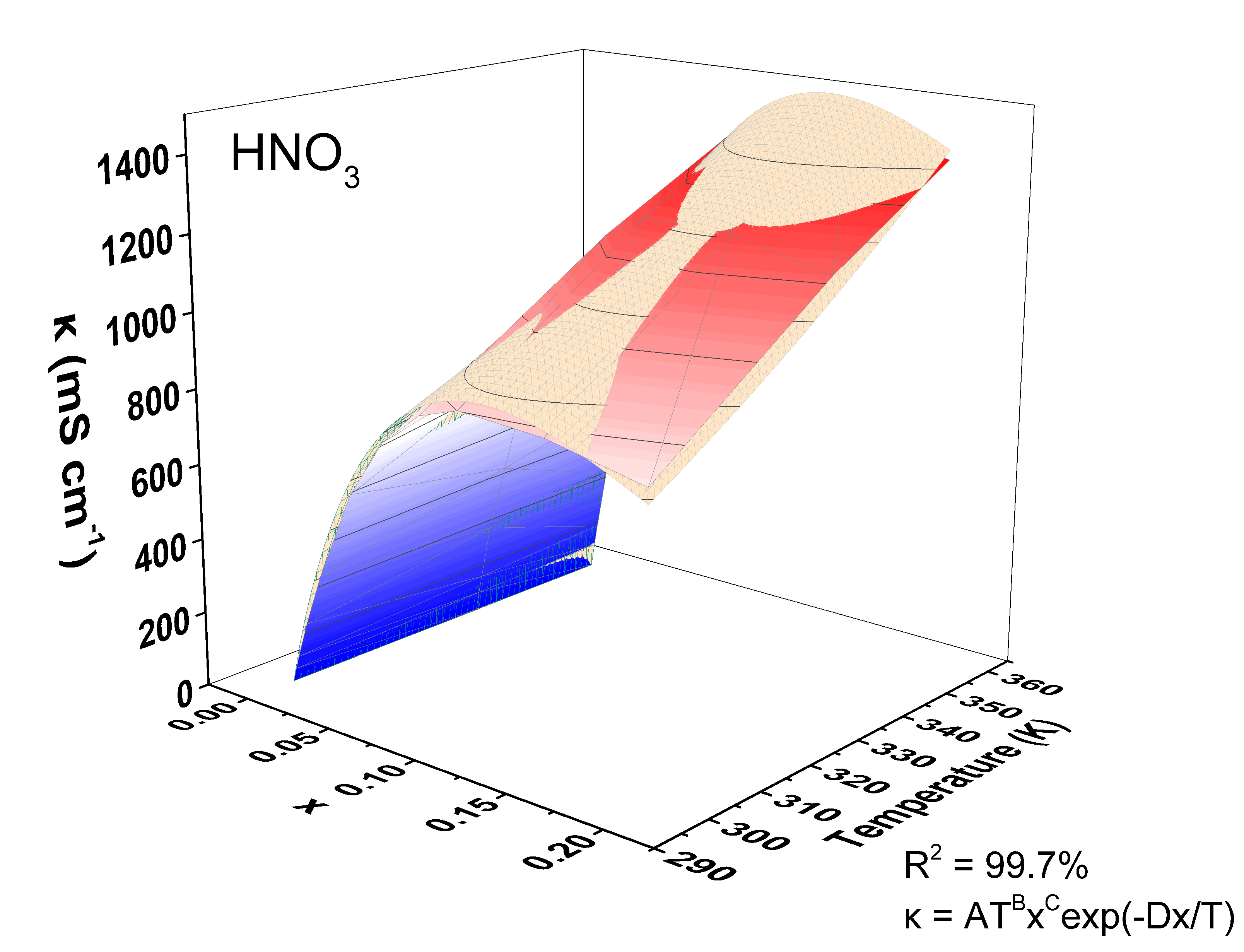

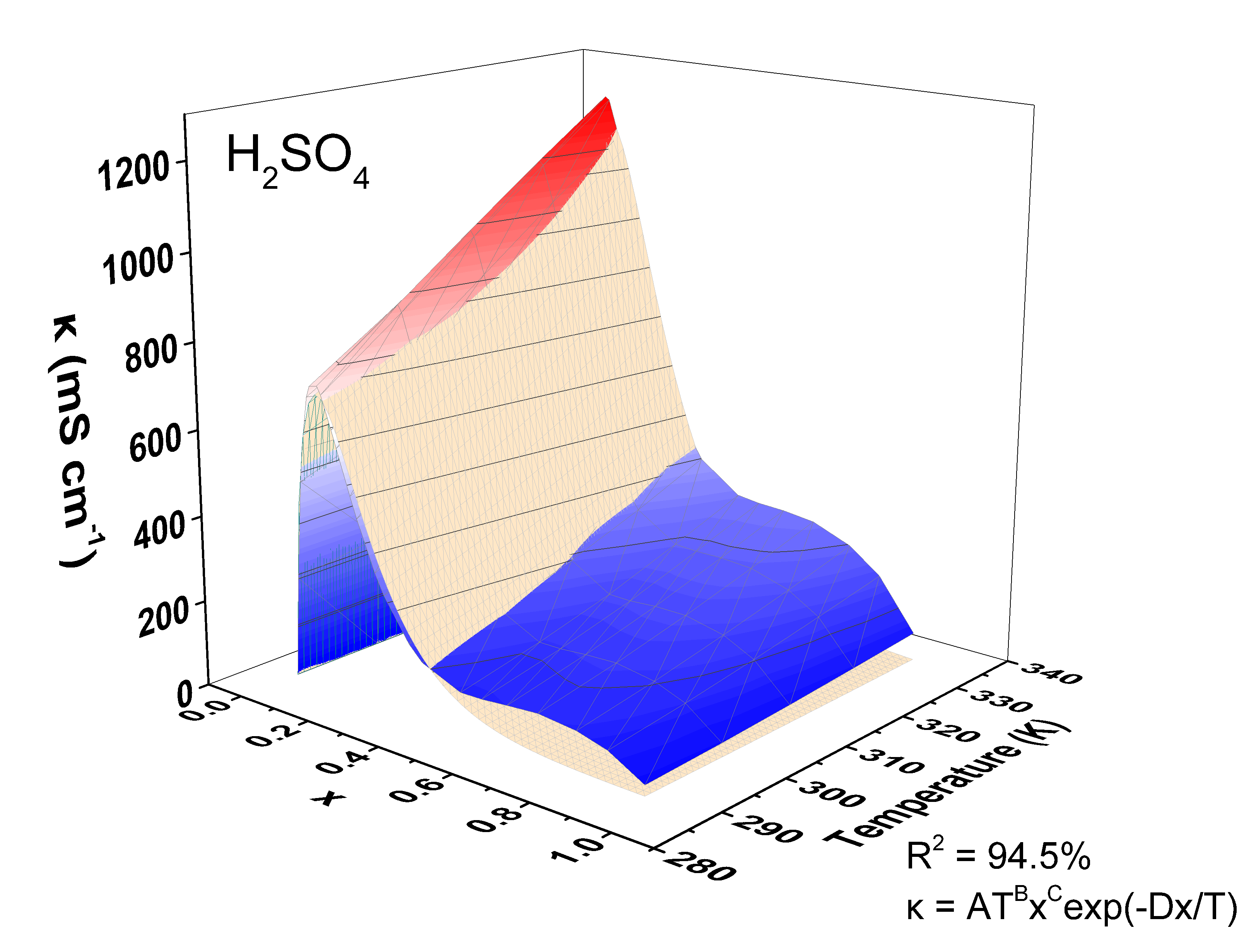

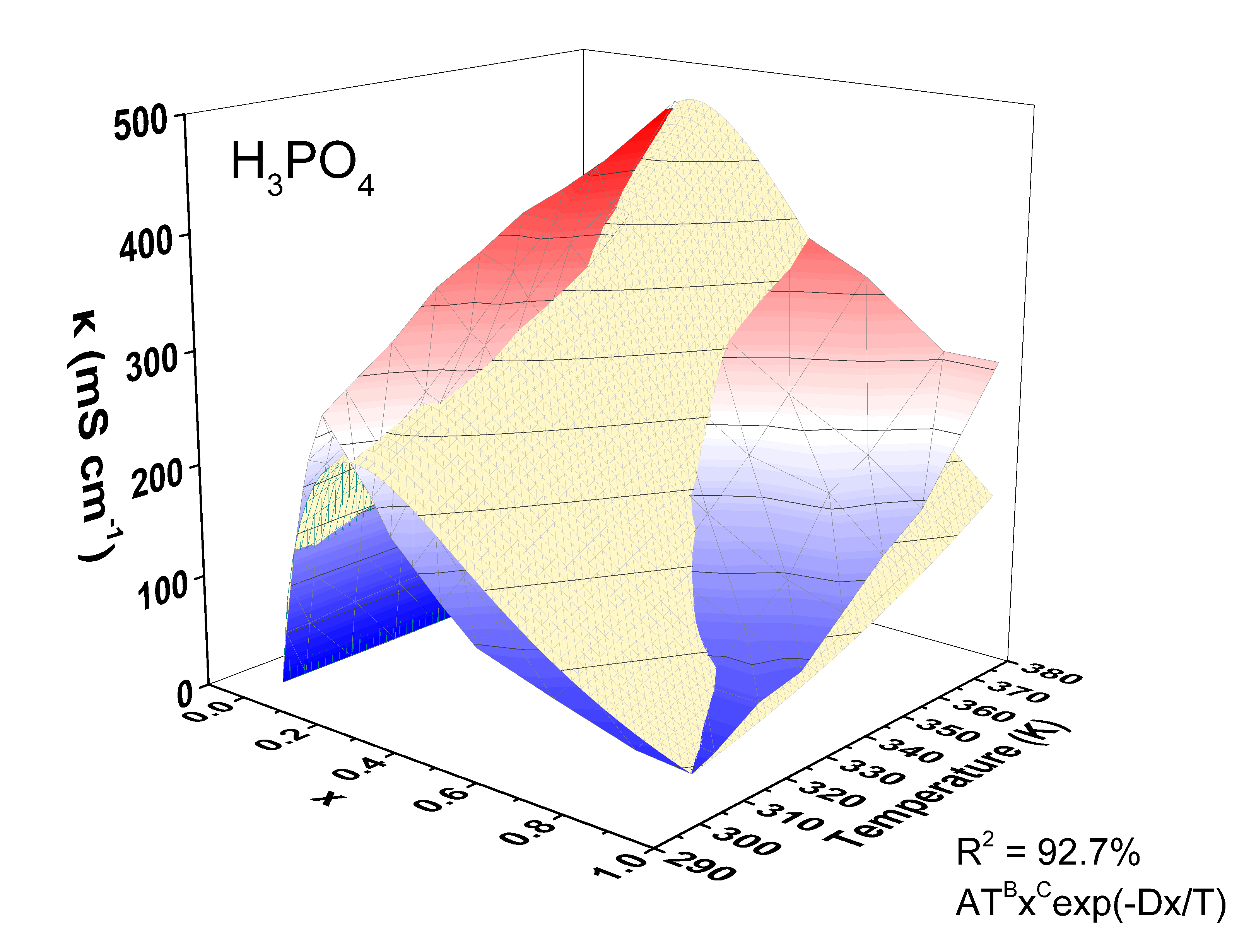


**Fig S.4:** Fitting of k vs x-T data for the four acidic aqueous solution against model found in ^[24]^


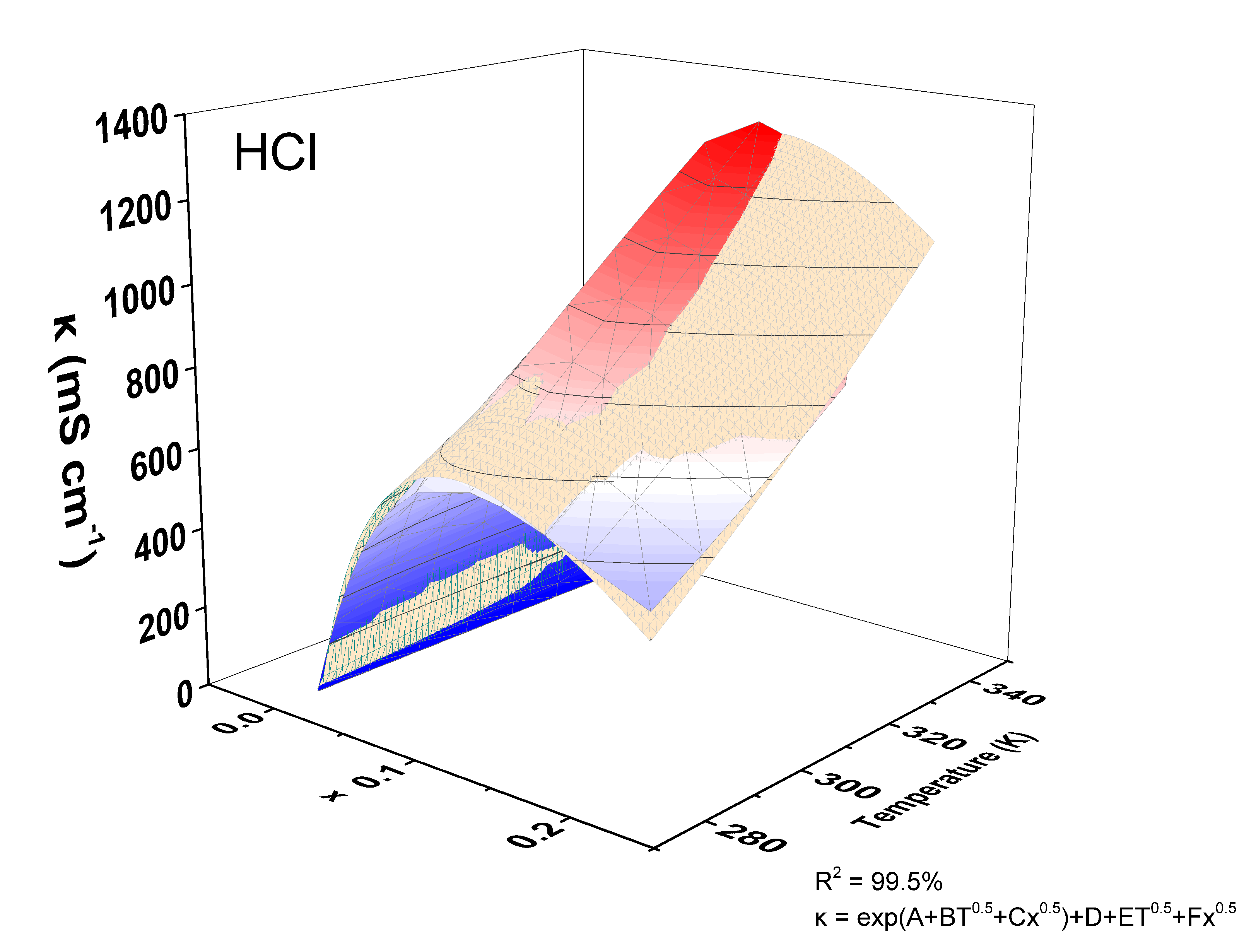

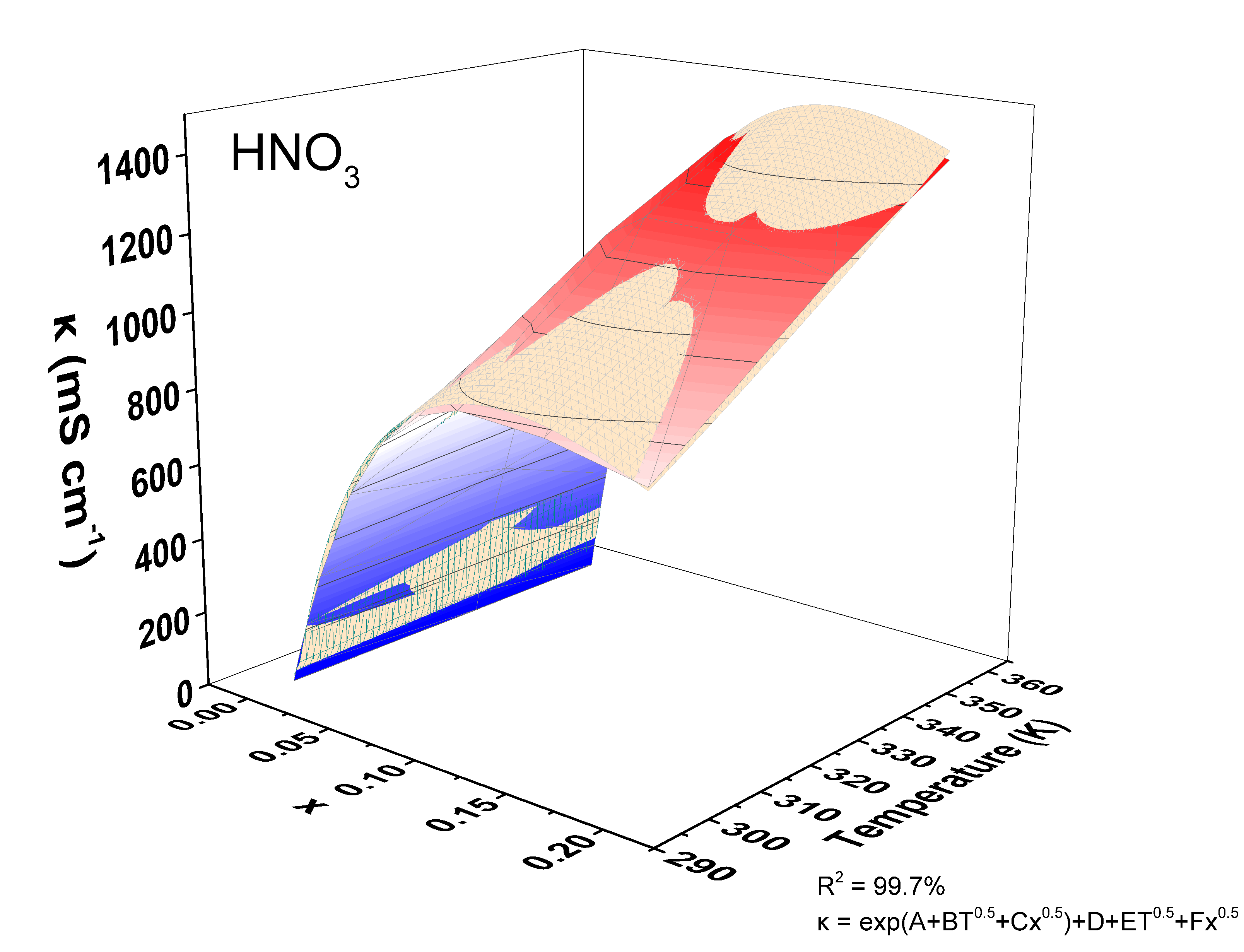

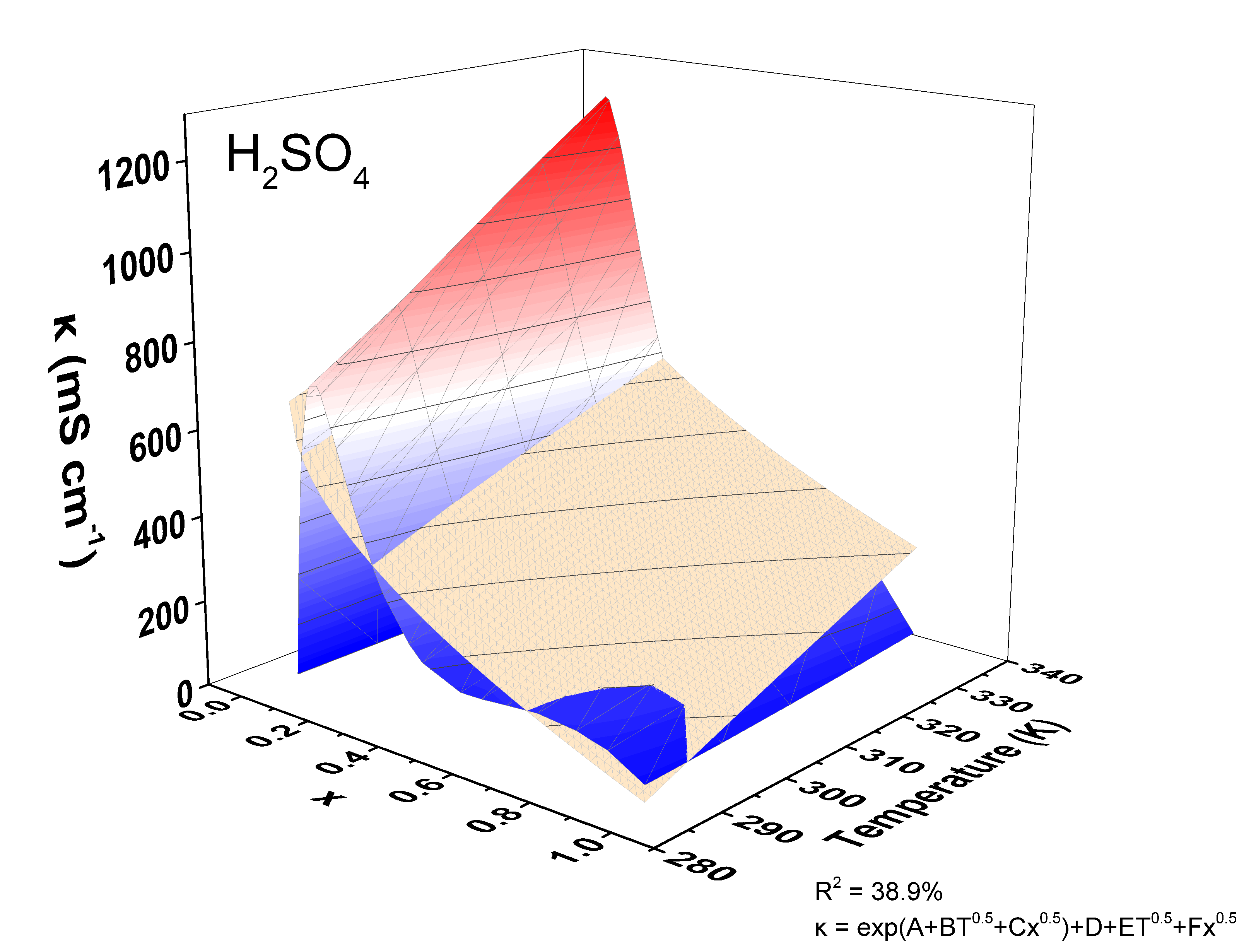

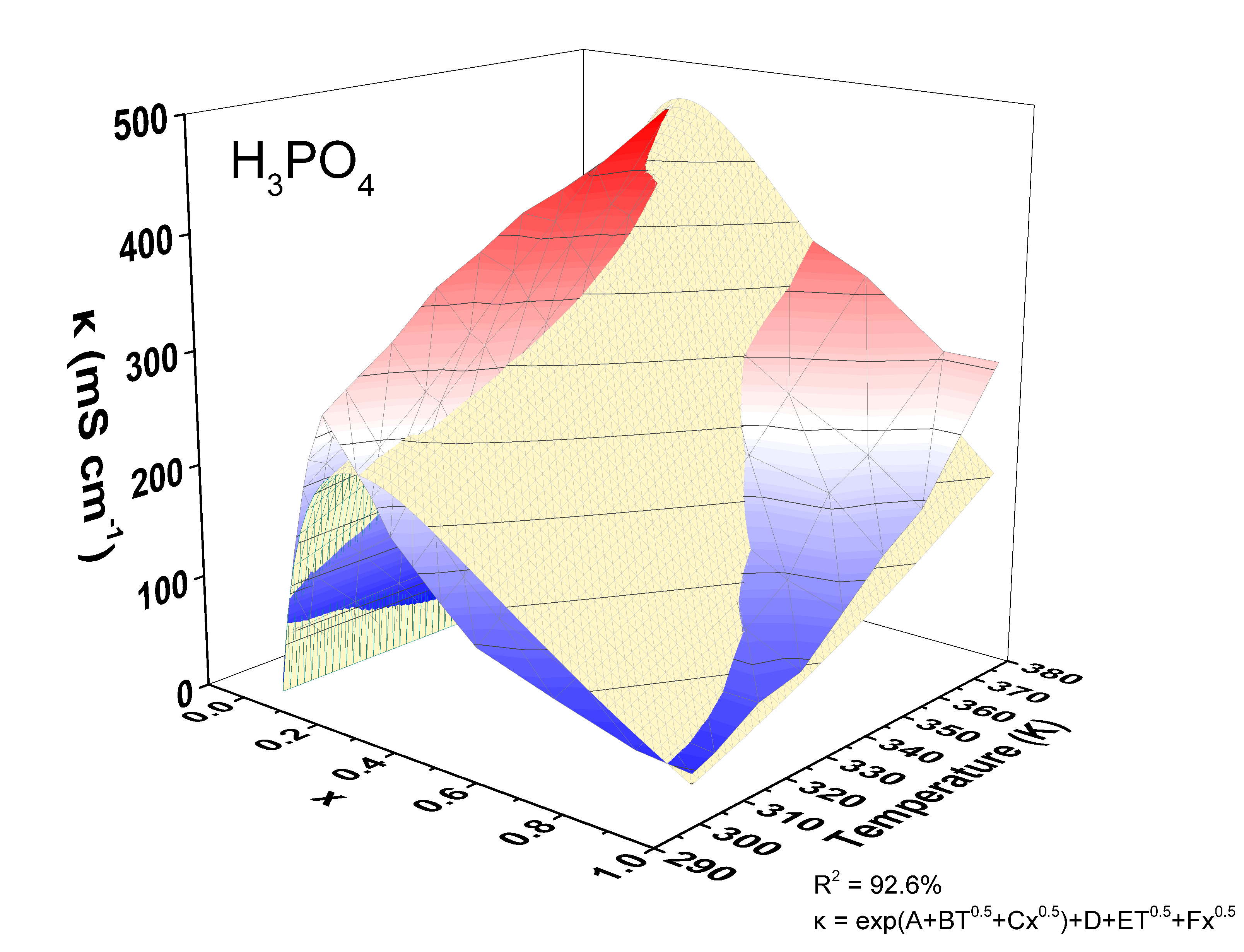


**Fig S.5:** Fitting of k vs x-T data for the four acidic aqueous solution against model found in ^[25]^


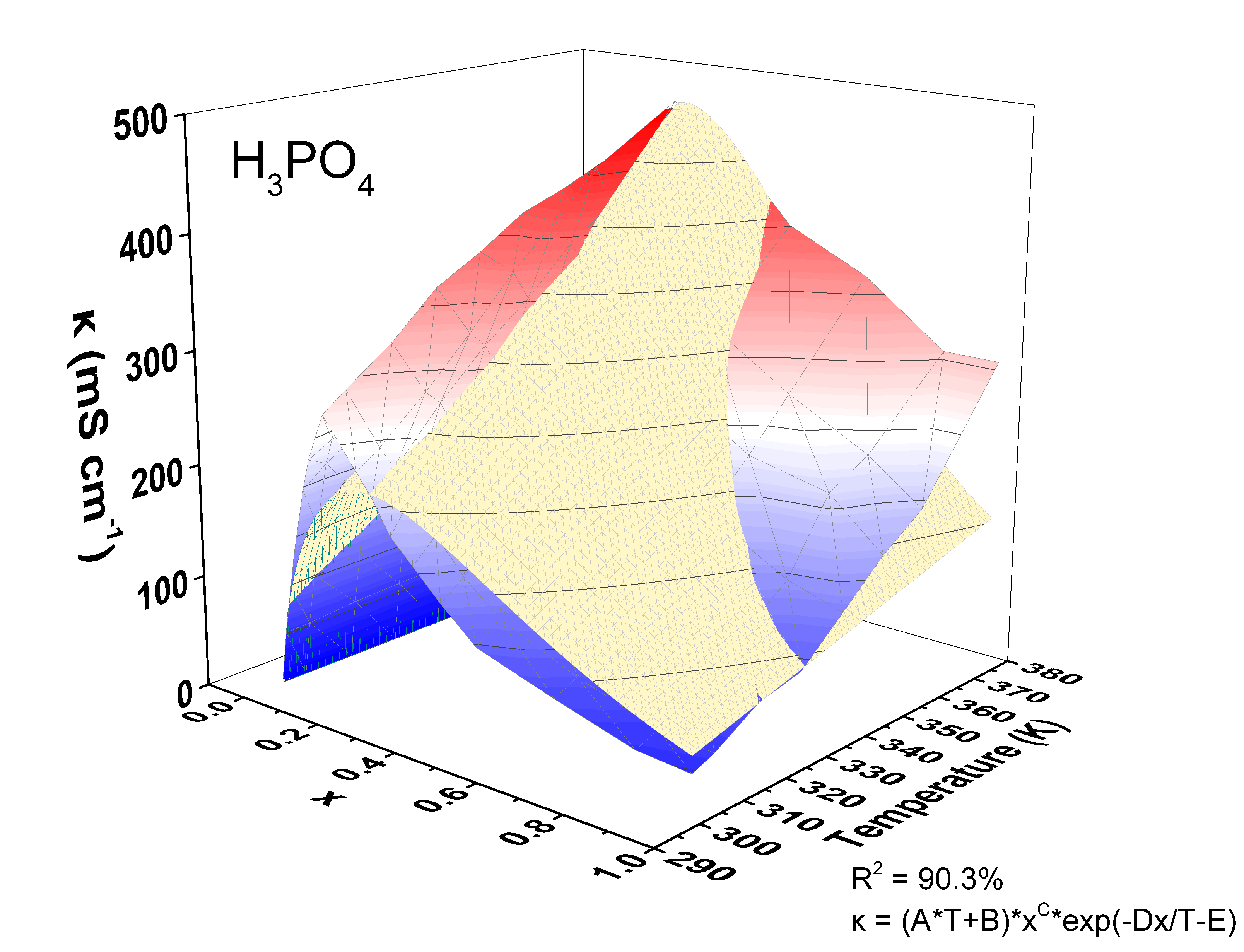

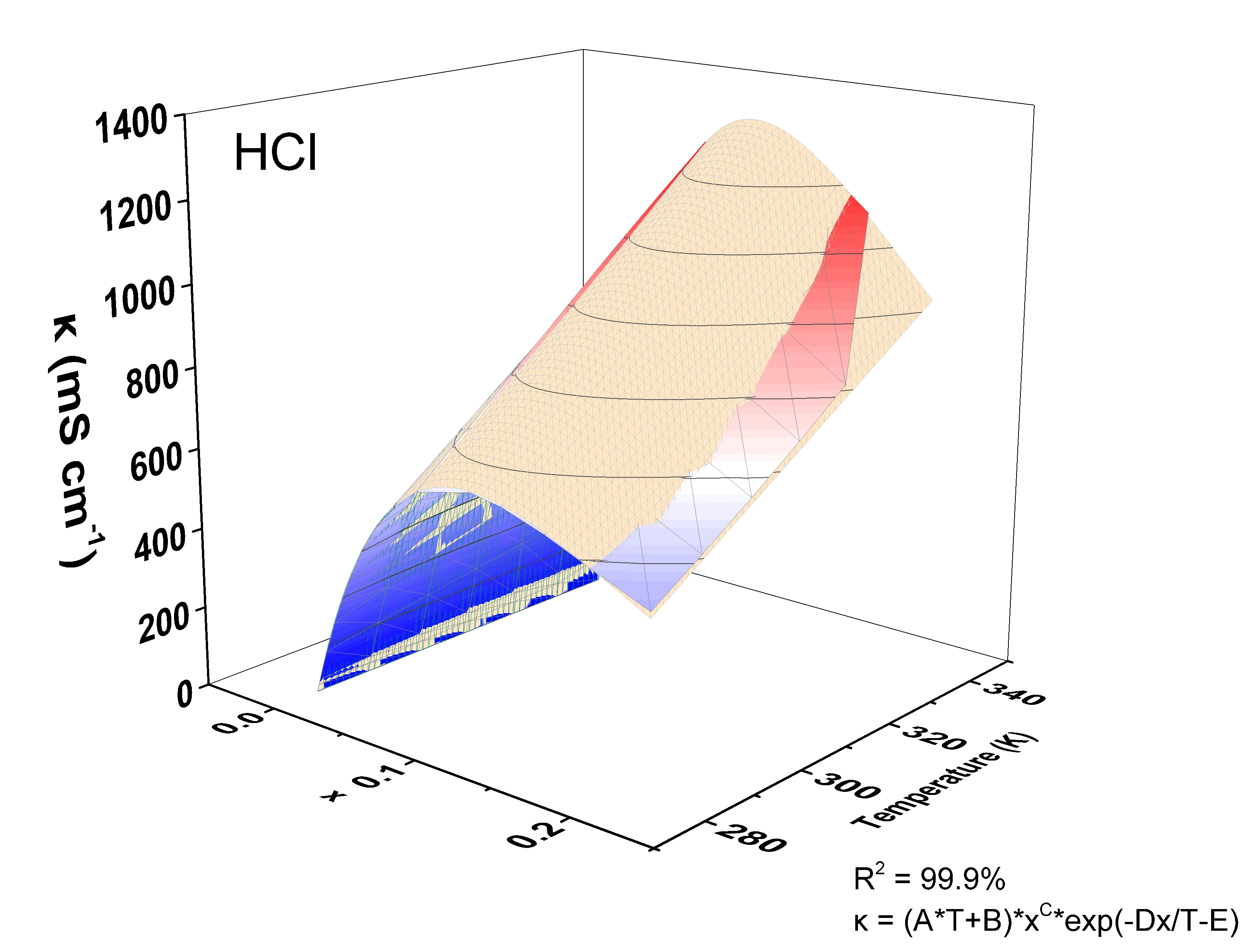

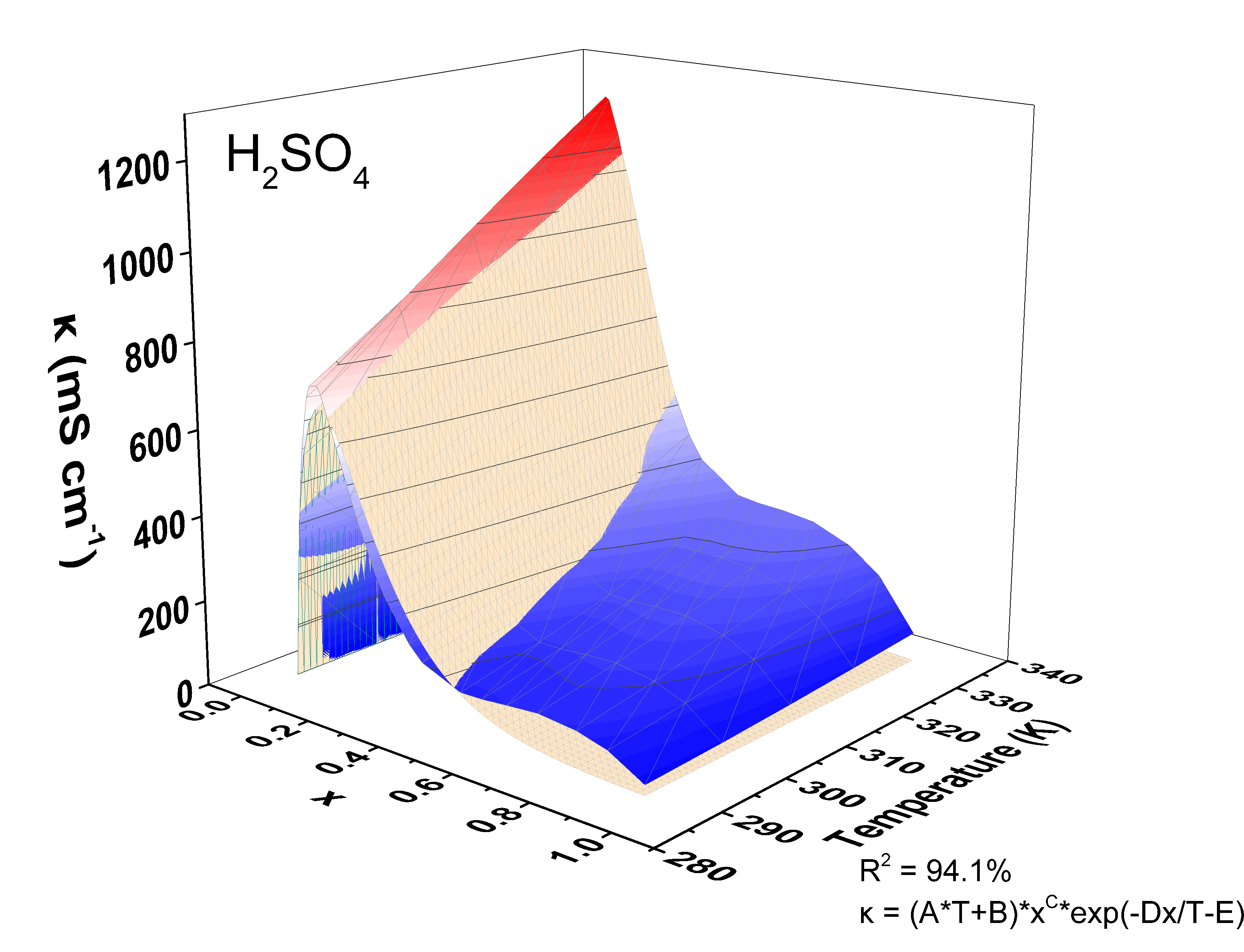

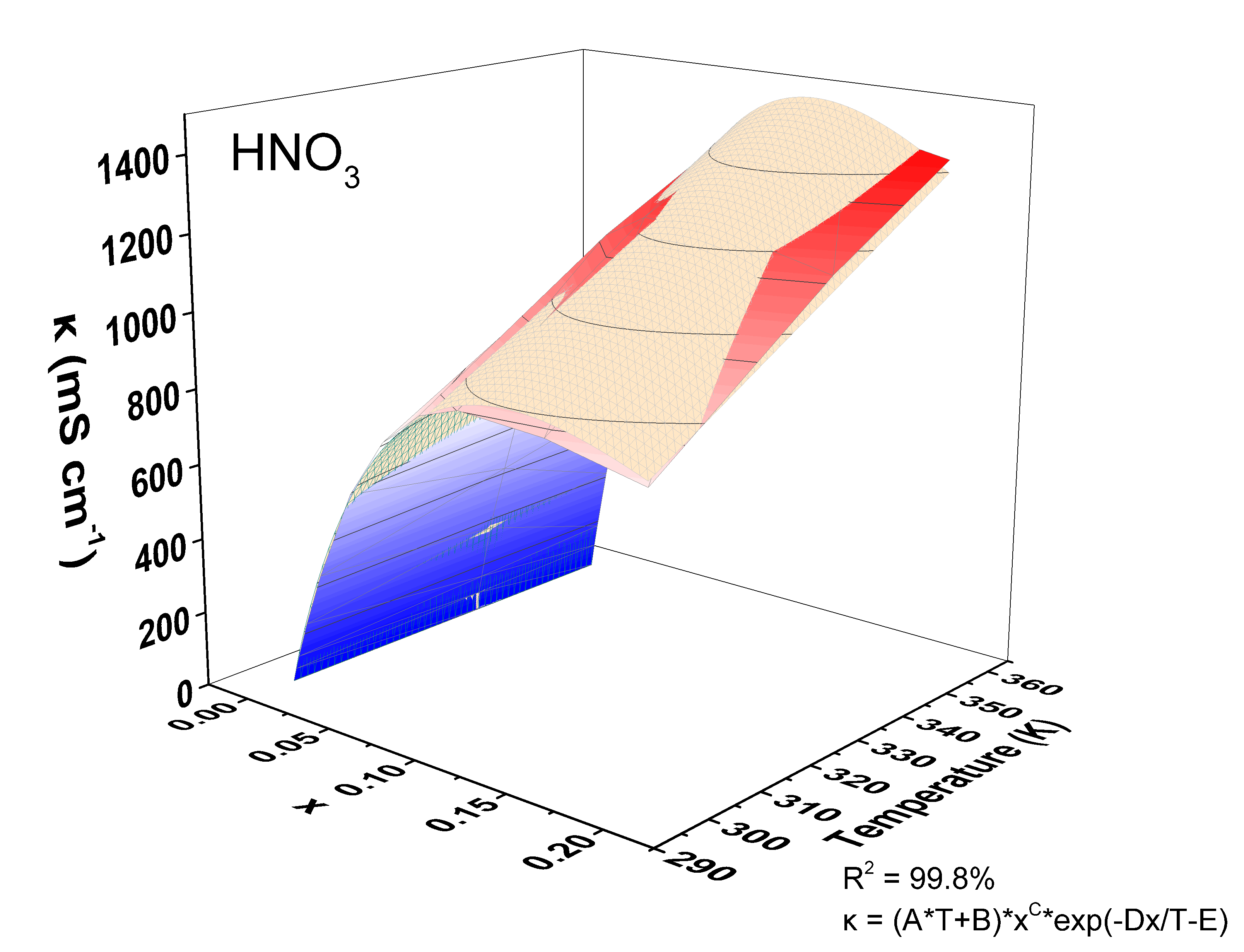

Supplement: Supplementary file 1 — Supplementary Information. [file 41598_2024_56552_MOESM1_ESM.docx]
